# Weight Perception Varies by Local Peer Body Size Norms Among US Adolescents

**DOI:** 10.1002/ajhb.70301

**Published:** 2026-06-30

**Authors:** Jennifer M. Cullin, Meagan M. Guilfoyle

**Affiliations:** ^1^ Department of Anthropology Indiana University Bloomington Indiana USA; ^2^ The Irsay Institute for Sociomedical Sciences Research Indiana University Bloomington Indiana USA; ^3^ Human Biology Program Indiana University Bloomington Indiana USA

**Keywords:** adolescents, biological normalcy, body size norms, United States, weight perception

## Abstract

**Introduction:**

Biological normalcy is a framework that examines relationships between statistical norms (e.g., variation, distribution) of biological traits and normative understandings of biology at the population level. We use this biocultural framework to test whether weight perception varies by local peer body size norms (BSNorm) in a nationally representative sample of US adolescents.

**Methods:**

We used 2023 Youth Risk Behavior Survey data. Weight perception was assessed as “underweight,” “about right,” or “overweight.” Local peer BSNorm was derived by calculating the proportion of peers with BMI ≥ 85th percentile within each locality. Linear regression tested the relationship between weight perception and BSNorm, controlling for sex, age, race/ethnicity, and BMI category (lower‐range: < 5th percentile, mid‐range: 5th–< 85th percentile, upper‐mid‐range: 85th–< 95th percentile, upper‐range: ≥ 95th percentile). Subgroup analyses by sex and BMI category were performed.

**Results:**

BSNorm was negatively associated with weight perception in the full sample (*β* = −0.60, *p* = 0.0005) and both sexes separately (females: *β* = −0.70, *p* = 0.0004; males: *β* = −0.50, *p* = 0.01). The association was primarily driven by adolescents in mid‐range (*β* = −0.58, *p* < 0.0001) and mid‐upper‐range (*β* = −0.76, *p* = 0.037) BMI categories. By sex‐specific weight categories, weight perception was negatively associated with BSNorm among females in the lower‐range BMI category (*β* = −2.37, *p* = 0.015) as well as males (*β* = −0.44, *p* = 0.014) and females (*β* = −0.76, *p* < 0.0001) in the mid‐range BMI category. There were no associations among adolescents in the upper‐range BMI category.

**Conclusion:**

Overall, weight perception decreased as local peer BSNorm increased. This association held for both males and females and appears to be largely driven by adolescents in the mid‐ and upper‐mid BMI ranges.

## 1 Introduction

Anthropological research has revealed relationships between statistical body size norms (BSNorm) and normative views of body size. For example, the past few decades have witnessed a global diffusion of anti‐fat attitudes as obesity rates continue to rise globally (Brewis et al. 2011), and US adolescents living in populations where it is more common to be thin demonstrated significantly higher anti‐fat bias compared to those living in areas where larger bodies are more common (Cullin 2021). Additionally, work on visual normalization suggests that regular exposure to higher obesity prevalence contexts may lead to a recalibration of the range of body sizes socially deemed “normal” in a population, essentially increasing the visual threshold for what is considered “overweight” (Robinson 2017). Although there are data demonstrating temporal shifts in weight perception as obesity prevalence increases, less is known about how contemporaneous geographic variation in local BSNorm of peers is related to weight perception among US adolescents. We use the biological normalcy framework to assess weight perception across 69 different US populations varying by statistical BSNorm.

Biological normalcy is a framework that investigates the ways in which statistical variation of a biological trait and normative cultural understandings of that trait are related to one another at the population level (Wiley 2023; Wiley and Cullin 2019). The main hypotheses of this framework are: (1) what people see around them (statistical norms) inform their beliefs about which phenotypes are ab/normal (the normative), and (2) these normative beliefs may change the population distribution of biological traits over time due to factors such as discrimination, privilege, differential mortality/morbidity, etcetera (Wiley 2023; Wiley and Cullin 2019). This study tests the first biological normalcy hypothesis by examining whether local peer BSNorm (i.e., statistical norm) predict weight perception (normative beliefs) in a nationally representative sample of US adolescents. We hypothesize that weight perception will vary depending on peer BSNorm in the local environment, and we predict weight perception will decrease as BSNorm increases. Additional exploratory analyses of this association are performed stratified by sex and BMI category. The second biological normalcy hypothesis is briefly addressed in the discussion with a review of literature on the relationship between weight perception and health outcomes.

## 2 Materials and Methods

### 2.1 Data

This analysis used data from the nationwide 2023 High School Youth Risk Behavior Survey (YRBS), a cross‐sectional survey conducted by the Centers for Disease Control every 2 years to monitor risk behaviors among students. These data are representative of public and private schools in the United States (Centers for Disease Control 2024b).

Age‐ and sex‐specific Centers for Disease Control (CDC) cut‐offs for BMI categories are used with alternative labels to avoid pathologizing language. We refer to these as lower‐range (under the 5th percentile), mid‐range (5th– < 85th percentile), mid‐upper‐range (85th– < 95th percentile) and upper‐range (95th percentile and above) BMI categories. Responses about weight perception were converted into a three‐point scale, with perceived overweight (“slightly overweight,” “very overweight”), “about right,” and underweight (“slightly underweight,” “very underweight”) corresponding to 1, 0 and −1. Students self‐reported height, weight, sex, age, race, and ethnicity. Race and ethnicity categories were collapsed into Hispanic, non‐Hispanic white, non‐Hispanic black, and other.

Local peer BSNorm were calculated as the proportion of respondents with BMI at or above the 85th percentile for each primary sampling unit (PSU) as a contextual measure of peer body size distribution. PSUs are deidentified indicators of the “counties, groups of smaller adjacent counties, or sub‐areas of very large counties” in which each participant's school is located (Centers for Disease Control 2024a).

### 2.2 Exclusion Criteria

Only students who responded to questions about height/weight, weight perception, age, sex, and race/ethnicity were included in the analysis. Eleven PSUs were excluded because they did not include a question about weight perception in the questionnaire. Two PSUs with small, unweighted sample sizes (*N* < 15) were excluded since prevalence estimates may be unreliable.

### 2.3 Statistical Analysis

This study uses SAS Enterprise Guide 8.6. per the YRBS data documentation (Centers for Disease Control 2024c), and survey procedures appropriate to complex survey sampling designs and weights are applied.

Descriptive analyses include chi‐squared tests for categorical variables and analysis of variance (ANOVA) for continuous variables. Linear regressions are applied to assess variation in weight perception by BSNorm. Multivariate linear regression models are constructed for the full sample and also stratified by sex and BMI categories. Models adjust for age and race/ethnicity, along with sex and BMI category when applicable.

BMI category (based on percentiles) is used as the indicator of weight status, as it reflects clinically (Kuczmarski et al. 2002) and socially (Robinson 2017) meaningful thresholds that shape weight perception and aligns with methods used in prior weight perception research (Foti and Lowry 2010). Sensitivity tests are run with BMI percentile in place of BMI categories and in within‐BMI category models to assess whether associations remain after accounting for variation in BMI percentile within BMI categories, and differences are noted.

### 2.4 Human Subjects Review Statement

The study used publicly available, de‐identified secondary data, thus institutional review board approval was not required.

## 3 Results

### 3.1 Participant Overview

The study sample included 10 660 students (weighted *N* = 12 945) across 69 peer groups (i.e., PSUs). BSNorm ranged from 9.8% to 51.2% and averaged 29.6%. About 30% of all students described themselves as “overweight” (see Table 1).

**TABLE 1:** Overview of sample characteristics for full sample and by BMI category.

	Full sample	BMI category				*p*
		Lower‐range	Mid‐range	Mid‐upper‐range	Upper‐range	
*N*	10 660	354	6767	1649	1890	
Weighted *N*	12 945	425.99	8559	1914	2047	
Age	16.0	16.3	16.0	16.0	16.0	0.0006
Sex (female)	46.3%	35.0%	48.0%	48.8%	39.2%	< 0.0001
Race/ethnicity						
Hispanic	29.2%	25.7%	27.3%	32.7%	34.6%	< 0.0001
Non‐Hispanic Black	13.6%	11.9%	12.3%	16.1%	16.9%	
Non‐Hispanic Other	10.0%	10.0%	10.5%	9.4%	8.2%	
Non‐Hispanic White	47.3%	52.5%	49.9%	41.8%	40.3%	
Weight perception	0.087	−0.694	−0.132	0.509	0.772	< 0.0001
Overweight (1)	30.0%	4.8%	13.4%	54.5%	81.9%	< 0.0001
About right (0)	48.7%	21.0%	60.0%	41.9%	13.5%	< 0.0001
Underweight (−1)	21.3%	74.2%	26.6%	3.6%	4.7%	

*Note:* *p*‐values are based on ANOVA for continuous variables and chi‐squared for categorical variables. Column percentages are reported.

### 3.2 Weight Perception and Local Peer BSNorm

**FIGURE 1:**
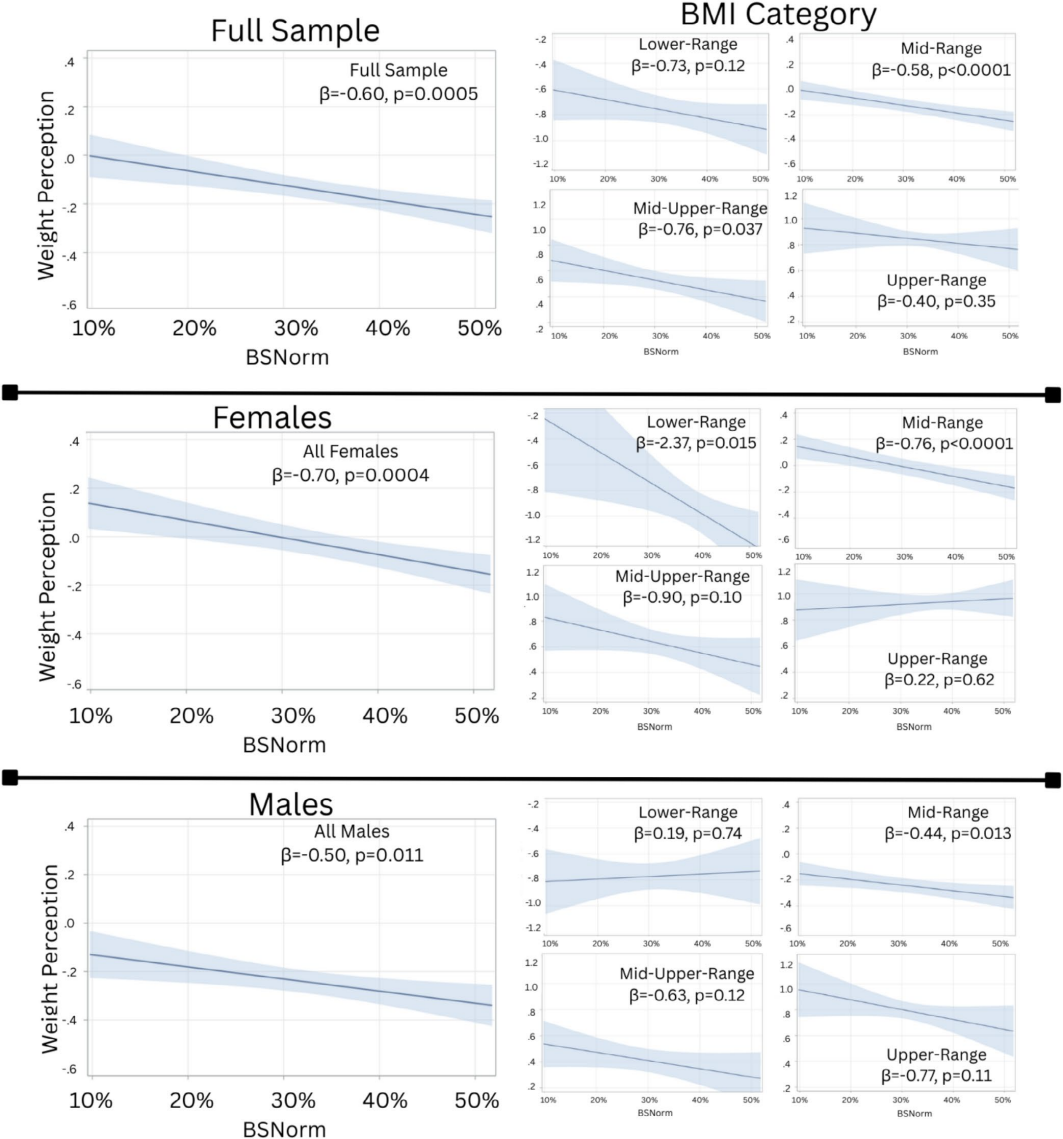
Probability of weight perception by local peer body size norms (BSNorm). Weight perception decreases as BSNorm increase, with variation in BMI categories for the total sample and across sex. BSNorm refers to the proportion of local peers with BMI ≥ 85th percentile. (1) The *y*‐axis range for each plot refers to weight perception and was adjusted per plot while maintaining a 1.0 scale for readability. (2) All models controlled for age and race/ethnicity; BMI category was controlled for in models that included all BMI categories, and sex was controlled for in models including males and females. (3) Plots were fitted to the average age within the subgroup and non‐Hispanic white race/ethnicity. In graphs that included both sexes, sex was included as numeric to fit the graph to the mean, and graphs that included all BMI categories were fitted to the mid‐range category.

BSNorm was inversely associated with weight perception in the full sample (*β* = −0.60, 95% CI: −0.91 to −0.28, *p* = 0.0005) as well as among females (*β* = −0.70, 95% CI: −1.07 to 0.33, *p* = 0.0004) and males (*β* = −0.50, 95% CI: −0.87 to −0.13, *p* = 0.01) separately. By BMI category, the association was primarily driven by youth in the mid‐range (*β* = −0.58, 95% CI: −0.84 to 0.32, *p* < 0.0001) and mid‐upper‐range (*β* = −0.76, 95% CI: −1.45 to 0.06, *p* = 0.037) BMI categories. By sex‐specific weight categories, weight perception was negatively associated with BSNorm among females in the lower‐range BMI category (*β* = −2.37, 95% CI: −4.22 to 0.52, *p* = 0.015) as well as males (*β* = −0.44, 95% CI: −0.78 to 0.10, *p* = 0.014) and females (*β* = −0.76, 95% CI: −1.09 to 0.42, *p* < 0.0001) in the mid‐range BMI category. Figure 1 illustrates the relationship between weight perception and BSNorm.

Sensitivity analyses for all models were performed using BMI percentile rather than BMI category. Most findings were robust except for two analyses, with non‐significant associations among males and youth in the mid‐upper BMI category.

## 4 Discussion

Local peer BSNorm predicted weight perception, with youth in the mid‐range BMI category and females showing the strongest associations. Specifically, as statistical BSNorm among local peers increased, weight perception decreased. This supports the visual normalization hypothesis as well as the first biological normalcy hypothesis (statistical norms influence normative views).

Regarding the second biological normalcy hypothesis (normative views influence statistical norms), public health literature has debated the issue of underestimating one's own weight, with some arguing it reduces motivation to lose weight, contributing to higher obesity prevalence (Yaemsiri et al. 2011), and others arguing it is associated with positive health behaviors and better health status (Blake et al. 2013). Importantly, research has linked high weight perception with worse mental health among US youth (Roberts and Duong 2013; Thurston et al. 2017) and worse cardiovascular health, independent of body fat percentage, among US adults (Cullin and White 2020).

Given that high weight perception is associated with poor mental and physical health, the results from this study suggest that US adolescents may be more vulnerable to the poor outcomes associated with high weight perception in peer contexts where larger bodies are relatively uncommon. This is particularly true for females and for adolescents falling into the mid‐range and mid‐upper‐range BMI categories. That said, adolescents with the highest BMIs (upper‐range) may be vulnerable to the health consequences of high weight perception regardless of peer body size context, which could reflect broader anti‐fat attitudes in the United States (Brewis et al. 2011). Future longitudinal research should test if cultural body size ideals in low versus high obesity prevalence contexts predict greater weight retention, weight gain, or morbidity.

Limitations of this study include cross‐sectional design (causality cannot be assessed), self‐reported height/weight for BMI calculations, and BSNorm being calculated for YRBS respondents (YRBS was designed to be nationally rather than locally representative, which may limit generalizability). Although interaction models suggested sex‐specific patterns in the relationship between BSNorm and weight perception, they could not be incorporated into study findings due to unstable parameter estimates.

## 5 Conclusion

Youth living in local environments with a greater proportion of peers with relatively high BMI are less likely to describe themselves as “overweight,” which suggests statistical BSNorm among peers may shape normative ideas about weight. This study makes an important contribution by demonstrating US adolescent weight perception may be a locally calibrated assessment based on social comparison to local peer BSNorm.

## Author Contributions

**J.M.C.:** conceptualization (equal), methodology (equal), supervision (lead), visualization (supporting), writing – original draft preparation (equal), writing – review and editing (lead). **M.M.G.:** conceptualization (equal), formal analysis (lead), methodology (equal), visualization (lead), writing – original draft preparation (equal), writing – review and editing (supporting).

## Funding

The authors have nothing to report.

## Ethics Statement

The study used publicly available, de‐identified secondary data, so institutional review board approval was not required.

## Conflicts of Interest

The authors declare no conflicts of interest.

## Data Availability

The data that support the findings of this study are available from the Centers for Disease Control (CDC) at https://www.cdc.gov/yrbs/data/index.html. These data were derived from the following resources available in the public domain: Youth Risk Behavior Surveillance System, CDC, https://www.cdc.gov/yrbs/files/2023/XXH2023_YRBS_Data.dat.
